# Retrospection of Research on Dragonfly and Damselfly (Odonata) During Past Fifty Years: A Bibliometric Review

**DOI:** 10.3390/insects16090945

**Published:** 2025-09-09

**Authors:** Gang-Qiang Fan, Shao-Zhao Qin, Chao-Xing Hu

**Affiliations:** 1 Qiandongnan Agriculture Science Institute, Kaili 556000, China; 2 College of Life and Health Science, Kaili University, Kaili 556011, China; 3 Provincial Special Key Laboratory for Developing and Utilizing of Insect Resources, Institute of Entomology, Guizhou University, Guiyang 550025, China

**Keywords:** Odonata, dragonfly, damselfly, biodiversity, conservation, research trends, bibliometric analysis

## Abstract

Dragonflies and damselflies (Odonata) play important roles in ecology and are used as model organisms in evolutionary studies, but a comprehensive overview of Odonata research has been lacking. Here, we conducted a bibliometric analysis of Odonata literature from 1974 to 2023 using Web of Science (Science Citation Index Expanded) data and CiteSpace software. We analyzed over 5000 publications to identify major contributors, research hotspots, and emerging trends in this field. The results show that annual research output increased greatly over time, with the United States being the most prolific country. Co-citation and keyword analyses revealed several dominant research themes, including life history and evolution, flight mechanics and predatory behavior, habitat and community ecology, biodiversity and conservation under environmental change, molecular phylogenetics, reproductive biology, and dragonfly wing mechanics. Citation burst analysis highlighted emerging fronts such as global Odonata species diversity and conservation efforts, advanced molecular phylogenetic studies, the use of dragonflies and damselflies as model species and bioindicators, and new investigations into wing kinematics. In conclusion, this study provides a quantitative retrospective of five decades of Odonata research, offering insights into the field’s development and guiding future research directions.

## Simple Summary

Dragonflies and damselflies are not just beautiful insects but also important indicators of environmental health. Scientists have published thousands of studies on these insects in the past fifty years, but a comprehensive overview of Odonata research has been lacking. We set out to analyze nearly 5000 scientific articles from the 1970s to today to find out what topics scientists have focused on and how this research has evolved. Our analysis showed that the number of studies has grown dramatically, especially in recent years, with the United States leading in publications, followed by countries in Europe, Asia, and Latin America. Researchers have explored many aspects of dragonfly and damselfly biology, including how they live and grow, how they fly and hunt, how they interact with their habitats, and how to protect them when their environment changes. We also identified new trends in recent research, such as efforts to catalog and conserve species worldwide, deeper genetic studies of their evolutionary relationships, and using these insects as natural “health monitors” of ecosystems. Understanding these patterns helps scientists and conservationists see what we know and what we still need to learn, guiding future studies that support environmental monitoring and biodiversity conservation.

## 1. Introduction

The insect order Odonata comprises approximately 6500 described species [according to the World Odonata List, updated semi-annually [https://www.pugetsound.edu/puget-sound-museum-natural-history/biodiversity-resources/insects/dragonflies/world-odonata-list (accessed on 1 May 2025)], traditionally grouped into three extant suborders: damselflies (Zygoptera), dragonflies (Anisoptera), and the monogeneric Anisozygoptera [1]. Although this species count is relatively moderate compared to other insect orders, odonates have become one of the most extensively studied insect groups. In ecology and evolutionary biology, Odonata are widely used as model organisms owing to their diverse ecological roles and adaptive traits [2,3,4]. Beyond their prominence in basic research, odonates are also recognized as valuable bioindicators of environmental health. For instance, because many odonate larvae thrive only in relatively clean, well-oxygenated waters (though tolerance to pollution varies by species) [5,6,7,8], they have proven to be sensitive indicators of water quality. Their high sensitivity to environmental changes, coupled with ease of identification and cost-effectiveness, has further facilitated the use of Odonata in monitoring broader phenomena such as climate change and other anthropogenic disturbances [9,10,11,12]. In agroecosystems, dragonflies and damselflies act as natural predators that contribute to the biological control of pest insects [13,14]. Furthermore, Odonata species serve as ecological links between aquatic and terrestrial habitats by transferring energy, nutrients, and microorganisms across ecosystem boundaries [15,16].

Reflecting this broad interest in Odonata, numerous scholarly works have synthesized various aspects of odonate biology. These include general overviews of Odonata biology [17,18,19,20] as well as specialized reviews focusing on voltinism [21], freshwater biodiversity [22], molecular phylogeny [8,23,24], taxonomic classification [25,26], sexual selection mechanisms [27], and color formation [28,29]. However, comprehensive bibliometric analyses that capture the overall progress of Odonata research remain scarce. Only a few such bibliometric studies have been conducted to date. For example, Bried and Samways [30] examined global trends in Odonata research within the context of freshwater applied ecology and conservation. Oliveira-Junior et al. [31] assessed general research patterns over recent decades. Miguel et al. [32] identified research gaps with an emphasis on applied ecology and conservation in Brazil. In addition, Palacino-Rodríguez [33] documented the historical evolution and current status of Odonata research in Colombia.

In order to address this gap, we employed a scientometric mapping approach (also known as bibliometric mapping) to quantitatively evaluate the Odonata research landscape. This method uses visual informatics techniques to depict the structure and dynamics of the field, thereby overcoming certain limitations of traditional narrative literature reviews. It provides an objective overview of historical research developments and helps identify future research priorities. Accordingly, the primary aim of our study was to conduct a comprehensive bibliometric analysis of the Odonata research literature in order to visualize and identify major contributors, critical research themes, and emerging trends. The specific objectives were: (i) to identify the most productive contributors at the country, institutional, and author levels; (ii) to identify and summarize the major research topics based on keyword co-occurrence patterns; and (iii) to highlight emerging research directions as indicated by recent keyword and citation trends in the field. These objectives are intended to provide insights into the current state of knowledge in Odonatology and to suggest potential future research directions based on the observed trends.

## 2. Materials and Methods

### 2.1. Data Acquisition

We selected the Science Citation Index Expanded (SCIE) database of Web of Science as the data source, due to its comprehensive coverage of natural sciences research. The search query used the keywords “dragonflies”, “dragonfly”, “damselflies”, “damselfly”, and “Odonata” (combined with OR operators). The search spanned literature from 1973 through 2023, and was conducted on 15 February 2024. We limited results to documents classified as articles or reviews, and to those published in English. After applying these criteria and cleaning the data, the search yielded 5042 bibliographic records available for analysis.

### 2.2. Data Analysis

We conducted a scientometric analysis using the CiteSpace software [Version 6.3.R3; available at https://citespace.podia.com (accessed on 24 April 2025)]. CiteSpace is a visualization tool designed to map knowledge domains and explore bibliographic networks. It helps in clarifying relationships between research topics, evaluating the state of a field, identifying hot research areas, and forecasting emerging trends by analyzing publication and citation patterns [34].

In a CiteSpace visualization, each node represents a specific entity (such as a cited reference). Nodes are depicted with “tree rings”, where the thickness of each ring reflects the frequency of co-citations involving that entity in a given time slice. The color of a node’s rings progresses from blue (indicating older co-citation links) to red (indicating more recent co-citation links), thereby illustrating the temporal evolution of citation connections [35]. Nodes with stronger co-citation links or longer time spans appear with thicker or multi-colored rings, highlighting their sustained influence over time.

CiteSpace groups related nodes into clusters based on their interconnectivity, revealing major thematic areas within the field. A node with a high betweenness centrality (BC) value serves as a bridge between different clusters, connecting otherwise separate research themes. Such high-BC nodes often signify influential publications or topics that link multiple research domains [36]. Identifying these bridging nodes helps highlight key focal points in Odonata research that integrate diverse subfields.

Additionally, CiteSpace detects emerging topics by identifying entities with strong citation bursts. A citation burst occurs when a reference or keyword experiences a rapid surge in citations over a short period, suggesting it marks an intellectual turning point or a “research front” in the field. CiteSpace employs Kleinberg’s burst-detection algorithm to capture these sudden increases in citation frequency [37]. The presence of a bursty node indicates a topic that has gained recent prominence and may represent an emerging trend in Odonata research.

### 2.3. Parameter Design

For our CiteSpace analysis, we configured the software parameters as follows. We set the time slicing to cover the years 1974–2023, using 1-year per slice to create annual snapshots of the data. Article titles, abstracts, and index terms (keywords) were selected as the term sources for detecting recurring words and phrases.

We chose the g-index as the node selection criterion in place of the traditional h-index. The g-index is an index that extends the h-index by giving additional weight to highly cited articles, thereby providing a more nuanced measure of overall citation performance [38]. In CiteSpace, the g-index is implemented with a scaling factor *k* to adjust the number of top-ranked items from each time slice that are included in the network. In this study, we set *k* = 10, which limited the network to a reasonable size while retaining the most influential references from each year. After generating individual yearly networks, we merged them into a single network covering the full 1974–2023 period. We then applied CiteSpace’s Pathfinder algorithm to prune the merged network, removing redundant or weaker links and thus simplifying the network structure for clearer visualization.

We further refined the analysis by assigning informative labels to the identified clusters. Each cluster was automatically labeled using the log-likelihood ratio (LLR) algorithm, which extracts salient noun phrases from the titles of publications citing the references in that cluster [35]. These cluster labels provide a concise description of the research theme represented by each group of nodes. Finally, to ensure consistency in our bibliometric data, we standardized institutional names. Different variants of a single institution’s name were unified under one official name. For example, entries such as “CNRS—Institute of Ecology & Environment (INEE),” “CNRS—National Institute for Earth Sciences & Astronomy (INSU),” and “Centre National de la Recherche Scientifique (CNRS)” were all consolidated and recorded simply as Centre National de la Recherche Scientifique (CNRS). This normalization of institution names helped in accurately identifying collaborative networks and productivity at the institutional level.

## 3. Results

### 3.1. Publication Output Analysis

**Figure 1:**
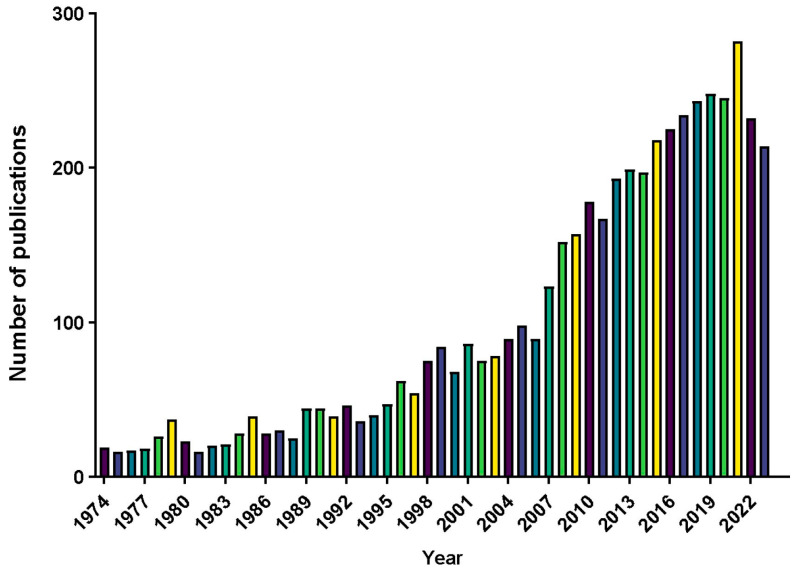
Number of publications in the field of Odonata research from 1973 to 2023 in the SCIE database (WoS).

In total, 5042 articles on Odonata were indexed from 1974 to 2023. The number of publications remained below 100 per year from 1974 to 2006, then showed a steady yearly increase up to 2022 (Figure 1). This indicates a substantial rise in research output in recent years.

### 3.2. Analysis of Research Countries

**Figure 2:**
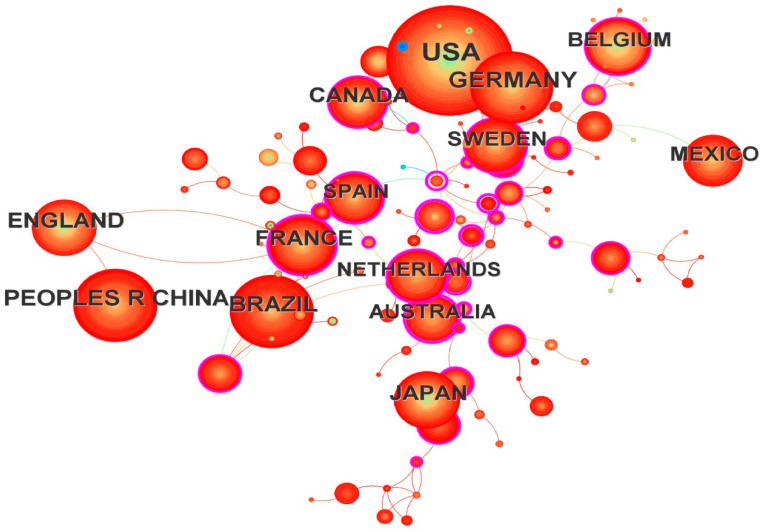
The network of co-country (125 nodes and 131 links) for the period 1973–2023.

Countries engaged in Odonata research were distributed worldwide. The top ten most productive countries were (in order): USA, Germany, China, Brazil, France, England, Canada, Japan, Belgium, and Mexico (Table 1). Collectively, these top ten countries accounted for about 74.18% of all publications, reflecting a highly uneven global distribution of research output. The USA alone contributed nearly three times as many publications as the second-ranked country, Germany [39]. Canada, although contributing fewer publications than most of the top ten countries, exhibited a high betweenness centrality (BC = 0.45) in the co-authorship network, indicating it acted as a key node connecting many other countries. The global co-country collaboration network contained 125 nodes and 131 links (Figure 2), a relatively low link-to-node ratio, suggesting limited collaboration among countries at the publication level.

### 3.3. Analysis of Research Institutions

**Figure 3:**
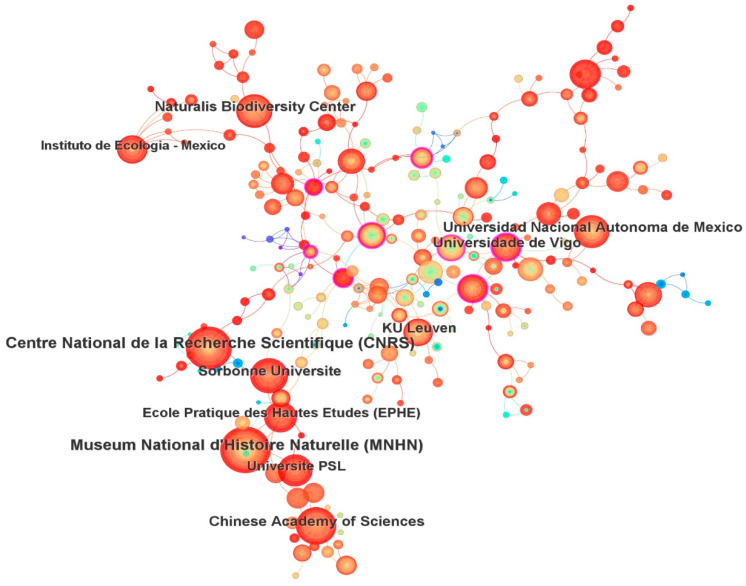
The network of co-institution (417 nodes and 350 links) for the period 1973–2023.

The top ten most productive research institutions were: Centre National de la Recherche Scientifique (CNRS), Museum National d’Histoire Naturelle, Chinese Academy of Sciences, Universidad Nacional Autónoma de México, Sorbonne Université, Naturalis Biodiversity Center, Universidade de Vigo, KU Leuven, Université PSL, and École Pratique des Hautes Études (Table 2). These ten institutions published a total of 1245 papers, accounting for 24.78% of all analyzed papers. Notably, eight of the top ten institutions are based in Europe, with the remaining two in Mexico and China. The co-institution network included 417 nodes and 350 links (Figure 3). The links between institutions were relatively few, indicating that there has been little inter-institutional cooperation in Odonata research.

### 3.4. Analysis of Research Authors

**Figure 4:**
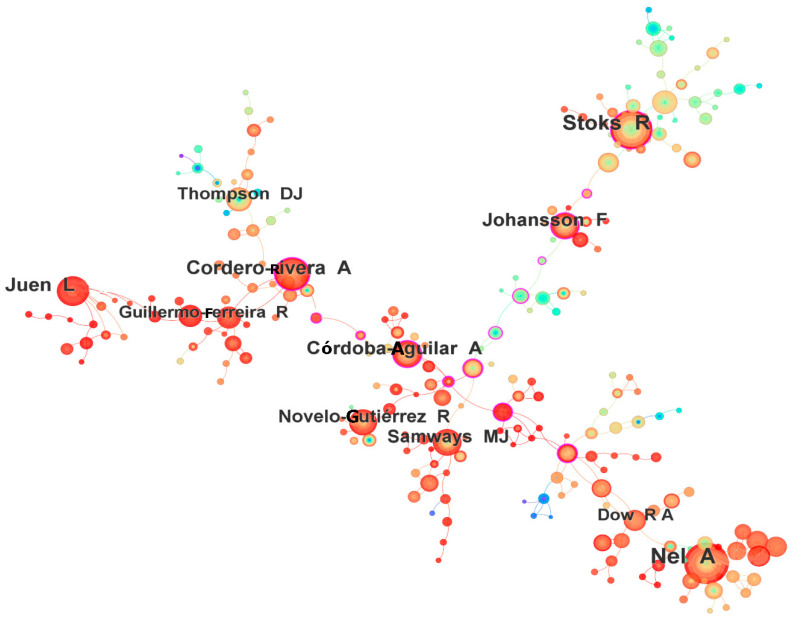
The network of co-author (553 nodes and 434 links) for the period 1973–2023.

The top ten most productive authors (and their affiliations) were: Nel A. (Sorbonne Université), Stoks R. (Katholieke Universiteit Leuven), Cordero-Rivera A. (Universidade de Vigo), Juen L. (Universidade Federal do Pará), Johansson F. (Uppsala University), Córdoba-Aguilar A. (Universidad Nacional Autónoma de México), Novelo-Gutiérrez R. (Instituto de Ecología, A.C.), Samways M.J. (Stellenbosch University), Thompson D.J. (University of Liverpool), and Dow R.A. (Universiti Malaysia Sarawak) (Table 3). The number of publications by these authors ranged from 42 to 164. The BC values of all these authors were low (each < 0.05), suggesting an absence of a single dominant research coordinator or topic in the field. The co-authorship network consisted of 553 nodes and 434 links (Figure 4). From this network, it appears that collaboration among researchers across different institutions was not extensive.

**Figure 5:**
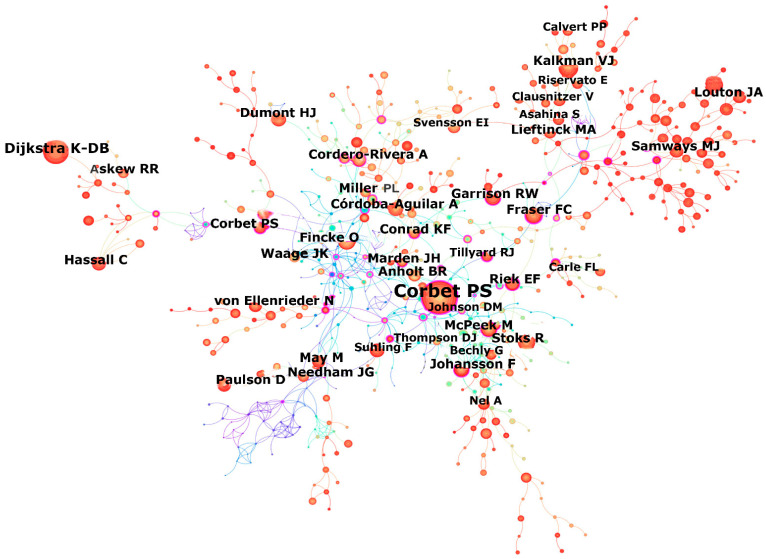
The network of co-cited author (657 nodes and 919 links) for the period 1973–2023.

The top ten most influential authors—those whose work was most frequently cited in Odonata research—were identified from the co-cited author analysis (Table 3, Figure 5). They included Corbet P.S., Dijkstra K.-D.B., Fincke O., Stoks R., May M., McPeek M., Johansson F., Kalkman V.J., Fraser F.C., and Garrison R.W. Only Stoks R. and Johansson F. appeared on both the most productive and most influential author lists. Corbet P.S. had the highest total citation count (1742), far exceeding the others on the influential authors list (whose citation counts ranged from 375 to 623). The co-cited author network (657 nodes, 919 links for 1973–2023) highlighted the key contributors and the structure of their collaborative links in the field of Odonata research (Figure 5).

### 3.5. Journal Co-Citation Analysis

The impact factor (IF) of each journal (as of 2023) is given in Table 4. As expected, general science journals such as Science and Nature have much higher IFs than specialized journals. These two journals were among the most frequently cited sources in our dataset, which may be due to their broad influence in the scientific community. Within the field, the journal Odonatologica had the highest number of total citations (2672), making it the most cited journal in Odonata research. The second most cited journal was Ecology (1421 citations). Notably, Odonatologica was also among the journals with the greatest number of Odonata publications, indicating that it is a central outlet for research in this domain. In general, the specialized Odonatology journals tend to publish the majority of papers on dragonflies and damselflies and also accumulate substantial citations within the field.

### 3.6. Analysis of Keywords

Keywords provide insight into the core content of the articles, and their analysis can highlight research frontiers in the domain. In our dataset, similar terms were unified (e.g., “dragonfly” and “dragonflies” were counted together) to avoid redundancy. Table 5 lists the top 20 keywords by frequency. The most frequent keyword was “Odonata” itself, which is expected given the focus of the study. Other highly frequent keywords included “dragonfly” and “damselfly”, reflecting that many studies specifically address these suborders. Several keywords pertain to taxonomy, such as “Insecta”, “Zygoptera”, “Anisoptera”, and “new species”, indicating a significant portion of research deals with taxonomic and systematic studies. The inclusion of terms like “conservation”, “biodiversity”, and “sexual selection” among top keywords suggests strong research interest in conservation biology and evolutionary ecology within Odonatology. Likewise, keywords such as “behavior”, “patterns”, and “evolution” point toward ecological and behavioral studies, while the appearance of “climate” as a frequent term reflects growing attention to the impact of climate and environmental change on Odonata.

**Figure 6:**
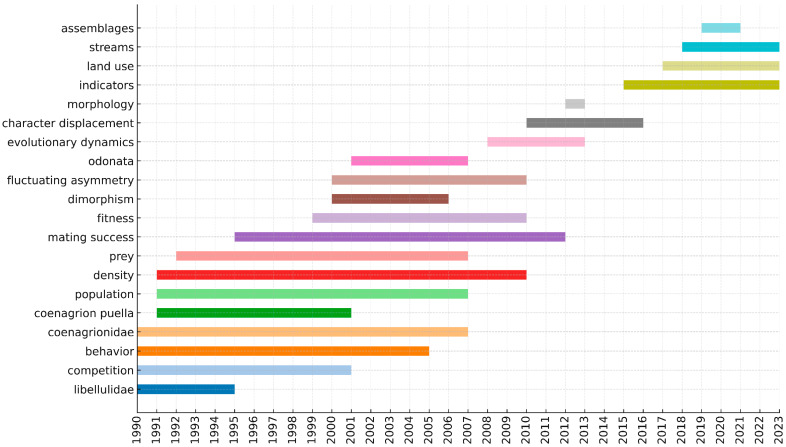
Chronology of citation-burst activity for the top 20 keywords (1990–2023).

Figure 6 shows the keywords with the strongest citation bursts, which can signify emerging topics. Based on these burst keywords, several potential research frontiers were inferred: (1) identification and protection of odonate species (e.g., terms like “Coenagrionidae”, “mating success”, “population”, and “fitness” suggest studies on species discovery, reproductive success, and population biology); (2) particular study habitats or regions (e.g., “streams” indicating a focus on lotic freshwater habitats); (3) biomechanics and behavioral ecology of odonates (e.g., “behavior”, “prey”, relating to predation and flight kinematics); and (4) effects of environmental change on Odonata (e.g., “land use” and “competition”, pointing to habitat alteration and interspecific interactions).

The timing of these keyword bursts reveals a clear shift in focus over the decades. For instance, taxonomy-related terms (e.g., “Odonata”, “Zygoptera”) dominate bursts in earlier years, whereas bursts in the 2000s–2010s include more applied terms like conservation, climate, and land use (Figure 6). This indicates that Odonata research has broadened from descriptive taxonomy toward conservation and environmental themes in recent years.

### 3.7. Analysis of Cited References

**Figure 7:**
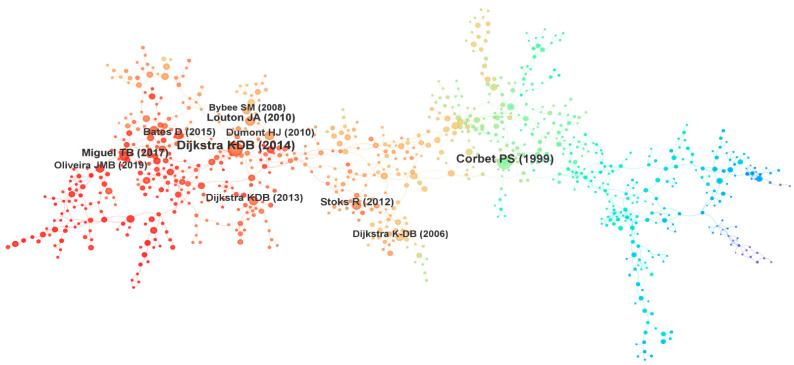
Co-cited reference network (1127 nodes and 1578 links) of bumblebee research from 1999 to 2023.

Co-citation analysis of references is a means to illustrate relationships among the literature in our dataset. The co-cited reference network (1127 nodes, 1578 links) displays the connections among key papers in Odonata research (Figure 7). Table 6 lists the top 10 most frequently cited references in our corpus. We summarize here the main content of these ten influential works:

(1) Corbet [18]—Dragonflies: Behavior and Ecology of Odonata (P.S. Corbet)—A comprehensive synthesis of dragonfly and damselfly behavior and ecology. This authoritative book systematically summarizes research on Odonata ecology and behavior and has served as a cornerstone reference in the field.

(2) Kalkman et al. [22]—Global diversity of dragonflies (Odonata) in freshwater—An overview of the global diversity and biogeography of Odonata, providing total species and genus counts per family across biogeographical regions.

(3) Clausnitzer et al. [40]—Odonata enter the biodiversity crisis debate: The first global assessment of an insect group—A landmark study that presented the first IUCN Red List assessment for a large sample of Odonata (1500 species, 26% of described species), highlighting conservation status and threats.

(4) Dijkstra et al. [41]—Redefining the damselfly families: a comprehensive molecular phylogeny of Zygoptera—A study reconstructing the damselfly family-level phylogeny based on extensive mitochondrial and nuclear DNA data, leading to taxonomic revisions in Zygoptera.

(5) Stoks and Córdoba-Aguilar [42]—Evolutionary ecology of Odonata: A complex life cycle perspective—A review that analyzes the evolutionary ecology of Odonata, emphasizing their complex life cycles and the insights these provide into ecological and evolutionary processes.

(6) Svensson et al. [43]—Female polymorphism, frequency dependence, and rapid evolutionary dynamics in natural populations—An empirical study demonstrating how male-female mating interactions in a color-polymorphic damselfly drive rapid evolutionary changes in morph frequencies across generations.

(7) Bybee et al. [44]—Molecules, morphology and fossils: A comprehensive approach to odonate phylogeny and the evolution of the odonate wing—A comprehensive phylogenetic analysis combining molecular and morphological data (including fossils) to understand dragonfly evolutionary relationships and wing evolution.

(8) Dijkstra et al. [25]—The classification and diversity of dragonflies and damselflies (Odonata)—A taxonomic work arguing for the conservation of certain family-group names and the retention of Epiophlebiidae as the sole family of the suborder Anisozygoptera.

(9) Dumont et al. [23]—A molecular phylogeny of the Odonata (Insecta)—A study estimating the phylogeny of Odonata based on nuclear ribosomal gene sequences, providing one of the first molecular frameworks for odonate phylogenetics.

(10) Oliveira-Junior et al. [45]—Neotropical dragonflies (Odonata) as indicators of ecological condition of small streams in the Amazon—A research article demonstrating that dragonfly assemblages are effective indicators of stream ecological health in the Neotropics, underscoring the need for enforcement of environmental protections.

**Figure 8:**
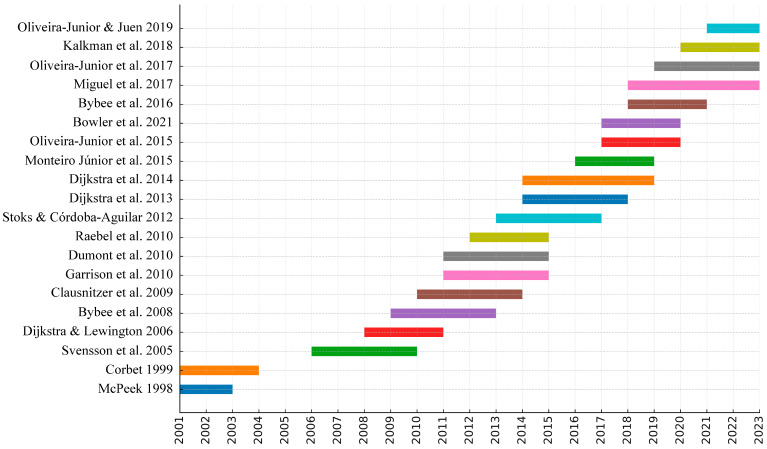
Chronology of the frequency of the 20 most-cited references that exhibit citation bursts (through 2023) [18,23,25,32,39,40,41,42,43,44,45,46,47,48,49,50,51,52,53,54].

Analyzing the co-citation burst data revealed that five of these top ten works also exhibit strong citation bursts, indicating they had periods of rapid increase in citations (Figure 8). Overall, the most influential references predominantly deal with global Odonata diversity and biogeography, conservation status, behavior and ecology, evolutionary and life-history studies, and molecular phylogenetics. Collectively, these highly cited works represent several of the major themes and influential contributions to Odonatology in the past half-century (Figure 8). However, they are not comprehensive—important areas such as insect flight mechanics, morphological variation, or citizen science initiatives do not appear in this top-cited list. In other words, the foundational knowledge base of Odonatology extends beyond what is captured by these most-cited publications.

## 4. Discussion

### 4.1. Publication Output Trends

Our results show that the annual output of Odonata research has increased dramatically over the past few decades, especially since the mid-2000s. This upward trend is consistent with previous bibliometric analyses of Odonatology literature, which also reported a marked rise in publications in recent years [31]. One likely driver of this growth is the expansion of publication venues in the 21st century. The launch of high-volume, broad-scope journals in the 2000s—such as PLOS ONE (established 2006) and Scientific Reports (2011)—provided new outlets that significantly boosted publication capacity for all fields, including Odonata research. Indeed, in our dataset three of the top 15 journals publishing Odonata papers were founded after 2000 (e.g., PLOS ONE, Scientific Reports, and Integrative and Comparative Biology). The availability of these new publishing venues coincided with and likely contributed to the rapid growth of Odonata publications. Moreover, the sustained increase in publication count suggests that dragonflies and damselflies have been attracting growing attention from the research community in general [31]. This heightened interest may stem from greater recognition of Odonata as model organisms in ecology and evolution, as well as concern for biodiversity conservation, which has put Odonata studies in the spotlight. However, it is important to note that this rise in Odonata publications parallels the overall growth in scientific literature during the past few decades. In other words, the expansion of Odonata research output partly reflects a general increase in research productivity across fields, rather than a trend exclusive to odonatology.

### 4.2. Geographic Distribution of Research

Odonata research output is highly skewed toward a few countries, with the United States being the clear leader. We found that the USA produced roughly three times as many publications as the next most productive country (Germany), a dominance that mirrors global trends reported in earlier studies [31]. The prominence of the USA (and, to a slightly lesser extent, countries like Germany, China, and Brazil) can be attributed to their longstanding research traditions and larger scientific infrastructures in entomology and ecology. For instance, the United States and Brazil have a broad, established history of Odonata research, which has been linked to robust academic communities and initiatives in those countries. Similarly, European nations such as Germany, as well as Asian countries like Japan and China, host leading odonatologists and extensive museum collections, reflecting a century-long legacy of dragonfly research in those regions [32].

In contrast, many other countries contribute relatively few publications on Odonata, indicating significant geographic disparities. This uneven distribution suggests that Odonata expertise and research activities are concentrated in certain “hotspots” (North America, Europe, parts of Asia and Latin America) while large parts of the world (including much of Africa, Asia, and Oceania) remain underrepresented. Such imbalance has also been observed in global assessments of Odonata research, which note that regions with long-term expert communities (e.g., North America, Europe) continue to dominate output, whereas areas lacking in resources or tradition (e.g., much of tropical Africa and Asia) produce fewer studies [31,32].

However, these publication counts are not adjusted for country population size or researcher base. For instance, the Netherlands (~18 million people) producing a similar order of magnitude of Odonata papers as a much larger country would indicate a higher per capita research activity. By contrast, populous nations like the USA (~340 million) or China (>1.4 billion) will naturally have higher absolute output. Therefore, direct comparisons should be tempered by the recognition that smaller countries can appear less productive in total output despite possibly high productivity per capita.

The analysis of collaboration at the country level further underscores this point. The relatively sparse connectivity in the international co-authorship network (Figure 2) indicates that cross-country collaborations in Odonatology are not as extensive as might be expected for a global field. Many studies appear to be carried out within single countries or through regional partnerships, rather than large international consortia. This fragmentation could be due to practical challenges (funding, language barriers) or simply because Odonata research often has a strong local focus (e.g., surveys of local fauna or region-specific conservation issues). Enhancing international collaboration could help bridge the gap for underrepresented regions. In fact, Oliveira-Junior et al. [31] have suggested that fostering partnerships between well-studied regions (e.g., Europe/North America) and under-studied areas (e.g., tropical countries) would build local research capacity and fill knowledge gaps. Initiatives such as joint surveys, training exchanges, and global networks (e.g., the Worldwide Dragonfly Association) are examples of efforts that could promote a more balanced global research landscape.

### 4.3. Institutional Contributions and Collaboration

Our bibliometric analysis revealed that European institutions are at the forefront of Odonata research output. Eight of the ten most productive institutions are based in Europe, led by centers in France (e.g., CNRS and the Museum National d’Histoire Naturelle) and including universities and museums in Belgium, the UK, and other countries. This European dominance reflects the continent’s rich tradition in entomology and taxonomy; Europe has a long history of dragonfly study dating back to the 19th and 20th centuries, which has translated into strong institutional expertise in the present day [32]. The remaining top institutions in our list—one from China and one from Mexico—show that other regions are also contributing significantly, likely as emerging hubs of Odonata research in Asia and Latin America. For example, the Chinese Academy of Sciences has invested in biodiversity research (including Odonata systematics and phylogeny), and Universidad Nacional Autónoma de México has a strong group of odonatologists focusing on Neotropical diversity and ecology. These institutions outside Europe provide important regional leadership and expertise, helping to broaden the global scope of Odonata research.

Despite the strong output from certain institutions, the collaboration network among institutions appears to be relatively weak. We observed that the co-institution network (Figure 3) had fewer links than nodes, and many of the top-producing organizations are not directly connected via co-authored papers. In practical terms, this means that many research groups operate in isolation or in small clusters rather than as part of a well-integrated global network. Limited inter-institutional collaboration could be a result of specialized research agendas or competition for funding and credit. It might also reflect the fact that Odonata studies often have a local or regional focus (e.g., surveying a country’s fauna or addressing local conservation issues), which naturally involves local institutions more than distant collaborations. However, increasing collaboration between institutions could yield benefits such as sharing expertise, standardizing methodologies, and tackling large-scale research questions (for example, continental biodiversity assessments or comparative studies across climates). As mentioned above, international and inter-institutional partnerships—for instance, between established research centers and institutions in biodiversity-rich developing countries—have been highlighted as a way to strengthen Odonatology globally [31]. Greater cooperation could facilitate comprehensive projects (like global monitoring programs or worldwide phylogenetic analyses) that no single institution could easily accomplish alone.

### 4.4. Authorship Patterns and Researcher Networks

The author-level analysis indicates a broad and decentralized contributor base in Odonata research. The list of prolific authors is international and spans multiple generations of researchers, from veteran odonatologists (e.g., A. Nel, M.J. Samways) to mid-career and younger scientists. Interestingly, only two individuals (R. Stoks and F. Johansson) are found in both the top ten by output and the top ten by citations. This suggests that being a highly prolific author does not necessarily equate to being highly cited in this field. In Odonatology, some authors have had an outsized influence through singular landmark works rather than sheer quantity of papers. For example, P.S. Corbet, who did not appear among the most prolific recent authors, amassed the highest citation count due largely to his seminal 1999 book on dragonfly behavior and ecology [18]. That comprehensive treatise has been extensively cited over the past two decades, underscoring how one foundational publication can elevate an author’s influence. On the other hand, authors like A. Nel or R. Stoks have built their reputations on a steady stream of publications, contributing incrementally to many areas of Odonata research. The distinction between prolific output and high citation impact hints at the dual nature of scientific contributions: some researchers drive the field forward by producing a large body of work, while others do so by producing a few transformative works that become standard references.

Another notable finding is the generally low betweenness centrality of the most productive authors in the co-authorship network. This network property suggests that no single researcher or tightly knit group serves as a central hub connecting all others. Instead, the Odonata research community is composed of multiple clusters or sub-communities that may be defined by geography, subdiscipline, or taxonomic focus. In practice, odonatologists often specialize (for example, some focus on taxonomy of specific families, others on ecology or physiology, etc.), and collaboration tends to occur within those sub-specialties rather than across the entire field. Our observation of many small clusters of collaboration aligns with the diverse array of research themes in Odonatology [31]. For instance, researchers working on fossil Odonata or phylogenetics might frequently collaborate with each other, but have fewer joint papers with those studying behavior or conservation, and vice versa. This thematic diversity inherently leads to a more distributed network of authors. Such a pattern is not unique to Odonata; in many fields of biology, specialization can limit the extent of collaboration among all top experts. Nonetheless, there is a degree of integration provided by certain influential figures—for example, authors like R. Stoks or F. Suhling (noted as influential in citations) have worked on broad topics like evolutionary ecology and may collaborate across subfields. Overall, the author network analysis highlights that Odonata research benefits from a wide base of contributors spread across different topics and regions, rather than being dominated by a few key players. This pluralistic structure may foster a rich variety of perspectives and approaches within the field, even if it means that coordinating large collaborative efforts is more challenging.

### 4.5. Journals and Research Dissemination

Our analysis of journals underscores the central role of specialized outlets in Odonata research. Field-specific journals such as Odonatologica and the International Journal of Odonatology have published the largest share of dragonfly and damselfly studies in the past fifty years [31]. These journals, dedicated to Odonatology, also garnered the highest citation counts within the field, reflecting their importance as repositories of Odonata knowledge. For example, Odonatologica not only contributed a significant number of articles but also was the single most cited journal in our dataset, indicating that papers published there are frequently referenced by other Odonata researchers. Such dominance of specialized journals is expected in a niche field, as they provide a focused platform for the community to publish new findings, whether they are species descriptions, ecological studies, or behavior observations.

In addition to the specialist journals, it is noteworthy that a few high-impact general science journals appear prominently in the co-citation analysis. Classic publications in Science and Nature (as well as top-tier ecology journals like Ecology) are among the most frequently cited sources in the Odonata literature. This suggests that certain discoveries about Odonata have had broad significance beyond the immediate field. For instance, studies of Odonata flight mechanics, neurology, or evolutionary biology that reveal general principles can be published in and cited from high-impact journals, hence their strong presence in citation networks. The influence of Science and Nature also indicates that when Odonata studies intersect with globally relevant themes (such as biodiversity loss or climate change), they reach a wider audience. This is exemplified by works like Clausnitzer et al. [40] on the global conservation status of Odonata and Thomas et al. [55] on climate-induced range shifts (though the latter is not in our top ten, such studies are often published in marquee journals and cited in Odonatology contexts). The representation of both specialized and general journals in the citation analysis highlights a dual publication strategy in the field: most Odonata-focused research is communicated within dedicated journals for the benefit of specialists, but the most far-reaching findings are disseminated in high-impact venues, ensuring that dragonflies and damselflies contribute to, and draw from, broader scientific discussions.

### 4.6. Research Themes from Keywords and Emerging Frontiers

The keyword analysis provides insight into the prevailing research themes and emerging trends in Odonata research. The prominence of core taxonomic terms (“Odonata”, “Zygoptera”, “Anisoptera”, and “new species”) among the keywords is unsurprising, as our search query explicitly included these group names. This result primarily reflects the focus on taxonomy and species discovery inherent in the literature retrieved, aligning with the long-standing efforts in odonate classification and species documentation over the past fifty years.

At the same time, the prominence of keywords such as “behavior”, “patterns”, “evolution”, and “sexual selection” indicates a strong interest in ecological and evolutionary questions. Many researchers have used odonates as model organisms to study behaviors (e.g., territoriality, mating) and evolutionary processes (e.g., sexual selection, life-history tradeoffs), which is reflected in the frequent mention of these terms. For example, sexual selection in Odonata, including phenomena like male-male competition and female polymorphism, has been a recurrent topic [43], aligning with the presence of “sexual selection” among the top keywords.

Conservation and environmental science themes are clearly evident as well. The inclusion of “conservation” and “biodiversity” among top keywords highlights that a considerable body of work is aimed at understanding and protecting odonate diversity. This ranges from studies assessing species’ extinction risk (as in the IUCN global assessment [40]) to those using dragonflies as bioindicators of ecosystem health. In fact, terms like “climate” and “land use” appearing in the burst keywords suggest an increasing focus on how environmental changes affect Odonata. Recent research has started to document the impacts of climate change on dragonfly populations (e.g., shifts in distribution and genetic diversity), as seen in studies of population genomics under climate stress [12]. Likewise, “land use” and “competition” emerging as hot topics reflect growing interest in how habitat alteration and species interactions (including invasive species or interspecific competition) influence odonate communities. These areas are critical for conservation, given that many Odonata species are sensitive to wetland degradation and climate fluctuations [31].

The burst analysis of keywords (Figure 6) points toward several emerging research frontiers in Odonatology. One notable emerging frontier is a renewed focus on species discovery and conservation. This is evidenced by burst keywords representing specific taxonomic groups (e.g., “Coenagrionidae”), which suggest intensified taxonomic surveying efforts, and by bursts of terms related to fitness and population dynamics, reflecting an increasing research emphasis on population ecology and species viability. Together, these trends align with the push for more comprehensive biodiversity surveys and Red List assessments of odonate in biodiverse regions, as well as studies on population ecology and genetics that can inform conservation strategies. Another emerging area is the detailed study of dragonfly flight mechanics and associated predator-prey dynamics, as evidenced by burst terms related to wing kinematics and prey. This suggests that researchers are increasingly examining how dragonflies capture prey and evade predators, aided by advances in high-speed videography and biomechanics. Such studies have yielded new insights into the aerial agility of dragonflies, effectively bridging biology and engineering [40,56].

Additionally, the keywords analysis highlights freshwater habitat research (“streams” as a burst term), which suggests intensified research on lotic systems and their odonate assemblages. This is in line with global freshwater biodiversity initiatives and the need to monitor water quality—dragonflies are increasingly used as indicator species in river and stream health assessments [31]. Finally, the effects of environmental change, such as habitat loss and climate warming, constitute a critical frontier. Studies like Oliveira-Junior et al. [45] have demonstrated Odonata’s value in signaling ecological conditions, and we anticipate more research assessing how Odonata populations respond to changing environments (e.g., shifts in phenology, range, or community structure under climate change).

In summary, our analysis of keywords over time indicates that the field has broadened from primarily descriptive, fundamental biology into more applied and interdisciplinary domains. For instance, earlier decades were dominated by taxonomic and basic biology terms, whereas recent years show a surge in keywords related to conservation biology, climate impacts, and biomechanics (see Figure 6 for keyword burst chronology). This broadening reflects global drivers of research interest—as concerns over biodiversity loss and climate change mount, Odonata research is naturally converging on these applied themes to provide answers and tools (such as bioindicator frameworks) for addressing environmental challenges.

### 4.7. Influential Works and Knowledge Base

The co-citation analysis of references offers a window into the foundational knowledge of Odonatology and the topics that have most engaged researchers. The most frequently cited works in our study (Table 6) include comprehensive reviews, global assessments and key empirical studies, which serve as common points of reference for many researchers. While these publications are widely influential, the field has grown through contributions from many researchers across diverse sub-disciplines. Notably, several of these influential works are extensive syntheses or datasets that have broad applicability:

Global Syntheses and Reviews: Corbet’s [18] book is a prime example of a work that aggregates and organizes decades of research on dragonfly and damselfly biology. Its enduring citation record attests to its value as a one-stop reference on Odonata ecology and behavior. Similarly, Stoks and Córdoba-Aguilar [42] provided an authoritative review of Odonata evolutionary ecology, focusing on their complex life cycles and how these influence ecological and evolutionary patterns. Such reviews distill knowledge in ways that continue to guide new studies, explaining their high citation impact.

Biodiversity and Conservation: A number of top-cited studies have dealt with assessing Odonata diversity on a large scale. Kalkman et al. [22] offered a comprehensive overview of global dragonfly diversity and biogeography, effectively setting a baseline for how many species are known and where major knowledge gaps lie. Clausnitzer et al. [40] tackled conservation, providing the first global evaluation of Odonata extinction risk and bringing dragonflies into the broader biodiversity crisis discussion. These works are highly cited because they supply essential data and arguments that underpin conservation priorities and further research—for instance, they are referenced whenever new species are described or when regional conservation statuses are debated.

Phylogeny and Taxonomy: Advances in the phylogenetic understanding of Odonata are well-represented among the influential references. Dumont et al. [23] and Dijkstra et al. [41] each made significant contributions by using molecular data to unravel the evolutionary relationships of dragonflies and damselflies. These studies helped update the higher-level classification of the order and often serve as a framework for comparative analyses. Additionally, Dijkstra et al. [25] addressed the classification and nomenclature within Odonata, reflecting the ongoing efforts by taxonomists to refine how we organize odonate diversity. The frequent citation of these works indicates that understanding “who is related to whom” among odonates is a central question that many studies touch upon, whether they are ecological, behavioral, or otherwise, since a robust taxonomy and phylogeny are prerequisites for comparative research.

Behavior and Evolutionary Dynamics: The influence of works like Svensson et al. [43] underscores the interest in Odonata as models for evolutionary processes. Svensson et al. [43]’s finding of rapid evolutionary change in damselfly morph frequencies is often cited in the context of studying natural selection and genetic polymorphism in the wild. Similarly, other behavioral ecology studies (e.g., on territorial behavior, mating systems) frequently cite classic Odonata research when drawing parallels or highlighting unique adaptations of dragonflies. The high citation counts of these works show that Odonata research has contributed significantly to broader concepts in ecology and evolution, such as frequency-dependent selection and life-history theory.

Functional Morphology and Physiology: Bybee et al. [44] represents the integration of fossil, morphological, and molecular evidence to explore wing evolution in Odonata. Its influence suggests that questions of form and function (in this case, how dragonfly wings evolved and the implications for flight) are of considerable interest. Odonata, with their ancient lineage and exceptional flight capabilities, provide important case studies for evolutionary biology, and works that illuminate these aspects become touchstones for both entomologists and evolutionary morphologists.

Odonata as Bioindicators: The inclusion of Oliveira-Junior et al. [45] among the top cited references highlights the applied side of Odonata research. This study demonstrated dragonflies’ utility in indicating the ecological conditions of freshwater habitats, strengthening the argument for using Odonata in environmental monitoring. Its influence is seen in the growing number of studies that cite it when adopting or discussing dragonfly-based indices of water quality or habitat integrity (indeed, several later works on different continents have built on Oliveira-Junior et al. [45]’s methodology).

Collectively, these influential works delineate the main pillars of Odonata research over the last fifty years: taxonomy and phylogeny (to catalog and understand the tree of life of odonates), ecology and behavior (to learn how these insects interact with their environment and each other), evolutionary biology (using Odonata to test ideas about natural and sexual selection, adaptation, and life cycles), and conservation science (assessing species’ status and using odonates as indicators for broader environmental health). The strong citation burst patterns observed for many of these references also suggest that Odonata research has had distinct phases of rapid development—for example, the surge in molecular phylogenetics in the 2000s, or the heightened focus on conservation in the late 2000s following the global assessment.

It is important to emphasize that many other areas of odonate research—such as studies on fluctuating asymmetry and developmental stability, the mechanics of dragonfly and damselfly flight, the micro- and nano-structural properties of cuticular tissues, contaminant bioaccumulation, and citizen-science–driven biodiversity surveys—are not represented among the top-cited references. For example, reviews of dragonfly flight integrate experimental and computational data to examine wing morphology, force generation and pursuit strategies [56]. Detailed anatomical studies show that wing flexibility arises from micro- and nano-structural features such as resilin in the cuticle [57]. Recent ecological work documents microplastic contamination in dragonfly larvae from rice-field ecosystems, and national monitoring programs engage thousands of citizen scientists to collect odonate larvae for contaminant analysis [58,59]. These examples illustrate that odonatology draws on diverse disciplines and continues to expand beyond the confines of its most highly cited publications. However, as shown in our co-authorship and co-citation network analyses, the Odonata research community is composed of multiple clusters or sub-communities defined by geography, subdiscipline or taxonomic focus (Section 4.4). Collaboration often occurs within these sub-specialties rather than across the entire field.

Limitations: It is important to recognize the limitations of our bibliometric dataset. Our analysis was confined to publications indexed in Web of Science (SCIE), which means a substantial body of Odonata research in non-indexed outlets is not represented. Many regional journals, monographs, and contributions by amateur odonatologists (who often publish outside high-impact journals) lie outside our data; for instance, if local journals and literature were included, the output and influence of certain communities (e.g., German odonatology) would likely be higher than shown here. Therefore, our results may underestimate the productivity or impact of researchers who publish in local languages or non-SCIE venues. In short, “science from Web of Science” is not the entirety of Odonata research, and our findings should be interpreted with that caution in mind. Because our analysis relies on Web of Science data and citation counts, it may also underrepresent influential work published in less widely indexed journals or in emerging fields with smaller citation bases; consequently, areas such as wing biomechanics, fluctuating asymmetry, microplastic bioaccumulation, and citizen-science initiatives can be less visible in citation rankings despite their growing significance. We have aimed to draw robust insights from the available data, but we avoid overemphasizing the results given these inherent coverage biases.

## 5. Conclusions

Separating the results and discussion of this bibliometric review allows us to clearly see what was found and what it means. The Results documented the patterns of publication output, key contributors (countries, institutions, authors), and thematic focus areas (keywords and highly cited works) in Odonata research from 1974 to 2023. The Discussion has interpreted those findings in light of existing literature and context: Odonata research has grown substantially and internationalized, yet remains unevenly distributed geographically; it encompasses a diverse array of topics, from taxonomy to conservation, which is reflected in a diffuse collaboration network; and the field’s knowledge base is built on a combination of specialized scholarship and cross-disciplinary insights that give dragonflies and damselflies an important place in ecological and evolutionary research at large. Every indication from our analysis points to a maturing field that not only looks back on a rich legacy (as evidenced by its influential literature) but also is moving forward on multiple frontiers to address contemporary scientific and environmental challenges [31]. Finally, by identifying recent hotspots and emerging themes, this review hints at potential future trajectories for Odonata research—particularly in conservation and climate-related studies, where we anticipate continued growth.

## Figures and Tables

**Table 1:** Top 10 productive countries.

Publications	Betweenness Centrality	Country
1003	0.07	USA
412	0.08	Germany
411	0	China
362	0.03	Brazil
301	0.29	France
300	0.03	England
280	0.11	Canada
248	0	Japan
236	0.11	Belgium
226	0	Mexico

**Table 2:** Top 10 productive institutions.

Publications	BC	Institution	Country
201	0.06	Centre National de la Recherche Scientifique	France
195	0.08	Museum National d’Histoire Naturelle	France
135	0.06	Chinese Academy of Sciences	Peoples’ R China
118	0.02	Universidad Nacional Autónoma de México	Mexico
115	0.09	Sorbonne Universite	France
110	0.04	Naturalis Biodiversity Center	The Netherlands
104	0.24	Universidade de Vigo	Spain
101	0.01	KU Leuven	Belgium
84	0.04	Universite PSL	Spain
82	0.02	Ecole Pratique des Hautes Etudes	France

**Table 3:** Top 10 most productive and top 10 most citation authors.

Ranking	Author	Publications	BC	Author	Citations	Betweenness Centrality
1	Nel A.	164	0.05	Corbet P.S.	1742	0.26
2	Stoks R.	123	0.10	Dijkstra K.-D.B.	623	0
3	Cordero-Rivera A.	80	0.15	Fincke O.	568	0.08
4	Juen L.	72	0.04	Stoks R.	426	0.02
5	Johansson F.	69	0.12	May M.	414	0.10
6	Córdoba-Aguilar A.	66	0.16	McPeek M.	397	0.12
7	Novelo-Gutiérrez R.	59	0.02	Johansson F.	390	0.12
8	Samways M.J.	58	0.05	Kalkman V.J.	379	0
9	Thompson D.J.	50	0.04	Fraser F.C.	378	0.33
10	Dow R.A.	42	0.09	Garrison R.W.	375	0.19

**Table 4:** Top 10 journals based on cited frequency.

Ranking	Journals	Cited Frequency	BC	Impact Factor (2023)
1	Odonatologica	2672	0.07	0.30
2	Ecology	1421	0.06	0.59
3	Science	1213	0.07	56.90
4	Nature	1193	0.08	64.80
5	Canadian Journal of Zoology	1185	0.08	0.869
6	American Naturalist	1157	0.05	2.067
7	Animal Behavior	1108	0	1.932
8	Hydrobiologia	1097	0.02	2.60
9	Evolution	1032	0.01	2.614
10	Behavior Economics	1030	0.02	2.161

**Table 5:** The top 20 keywords in terms of frequency and centrality.

Ranking	Frequency	Keywords	Ranking	Frequency	Keywords
1	757	Odonata	11	227	conservation
2	442	dragonfly	12	212	selection
3	413	body size	13	205	life history
4	398	evolution	14	198	biodiversity
5	362	insecta	15	187	damselfly
6	301	behavior	16	180	sexual selection
7	288	Zygoptera	17	161	Anisoptera
8	273	diversity	18	156	climate change
9	246	patterns	19	151	community
10	232	new species	20	140	assemblages

**Table 6:** The top 10 most cited references in terms of frequency and centrality.

ID	Frequency	Year	Title
1	494	1999	Dragonflies: behavior and ecology of Odonata.
2	288	2008	Global diversity of dragonflies (Odonata) in freshwater
3	284	2009	Odonata enter the biodiversity crisis debate: The first global assessment of an insect group
4	260	2014	Redefining the damselfly families: a comprehensive molecular phylogeny of Zygoptera (Odonata)
5	226	2012	Evolutionary Ecology of Odonata: A Complex Life Cycle Perspective
6	218	2005	Female polymorphism, frequency dependence, and rapid evolutionary dynamics in natural populations
7	149	2008	Molecules, morphology and fossils: a comprehensive approach to odonate phylogeny and the evolution of the odonate wing
8	143	2013	The classification and diversity of dragonflies and damselflies (Odonata)
9	126	2010	A molecular phylogeny of the Odonata (Insecta)
10	115	2015	Neotropical dragonflies (Insecta: Odonata) as indicators of ecological condition of small streams in the eastern Amazon

## Data Availability

No new data were created or analyzed in this study. Data sharing is not applicable to this article.

## References

[1] Newton L., Tolman E., Kohli M., Ware J.L. (2023). Evolution of Odonata: Genomic insights. Curr. Opin. Insect Sci..

[2] Serrano-Meneses M.A., Córdoba-Aguilar A., Azpilicueta-Amorín M., González-Soriano E., Székely T. (2008). Sexual selection, sexual size dimorphism and Rensch’s rule in Odonata. J. Evol. Biol..

[3] Monzó J.C., Verdú J.R. (2022). Effects of restoration and management of Mediterranean traditional water systems on Odonata alpha diversity: A long-term monitoring survey. Biodivers. Conserv..

[4] Wellenreuther M., Dudaniec R.Y., Lancaster L.T., Córdoba-Aguilar A., Beatty C.D., Bried J.T. (2023). Genomic insights into micro- and macro-evolutionary processes in Odonata. Dragonflies and Damselflies: Model Organisms for Ecological and Evolutionary Research.

[5] Kutcher T.E., Bried J.T. (2014). Adult Odonata conservatism as an indicator of freshwater wetland condition. Ecol. Indic..

[6] Golfieri B., Hardersen S., Maiolini B., Surian N. (2016). Odonates as indicators of the ecological integrity of the river corridor: Development and application of the Odonate River Index (ORI) in northern Italy. Ecol. Indic..

[7] Janssen A., Hunger H., Konold W., Pufal G., Staab M. (2018). Simple pond restoration measures increase dragonfly (Insecta: Odonata) diversity. Biodivers. Conserv..

[8] Guillermo-Ferreira R., Juen L. (2021). Odonate ethodiversity as a bioindicator of anthropogenic impact. Int. J. Odonatol..

[9] Bush A., Theischinger G., Nipperess D., Turak E., Hughes L. (2013). Dragonflies: Climate canaries for river management. Divers. Distrib..

[10] Bush A.A., Nipperess D.A., Duursma D.E., Theischinger G., Turak E., Hughes L. (2014). Continental-scale assessment of risk to the Australian Odonata from climate change. PLoS ONE.

[11] Silva L.F.R., Castro D.M.P., Juen L., Callisto M., Hughes R.M., Hermes M.G. (2021). Functional responses of Odonata larvae to human disturbances in Neotropical savanna headwater streams. Ecol. Indic..

[12] Tolman E.R., Bruchim O.R., Driever E.S., Jordan D., Kohli M.K., Montague L., Park J., Park S., Rosario M., Ryu J.L. (2023). Changes in effective population size of Odonata in response to climate change revealed through genomics. Int. J. Odonatol..

[13] Holt R.D., Lawton J.H. (1994). The ecological consequences of shared natural enemies. Annu. Rev. Ecol. Evol. Syst..

[14] Rahong P., Techakijvej C., Phalaraksh C. (2023). Predators as biocontrol agents of mosquito larvae in small and large habitats in Chiang Mai, Thailand. J. Vector Ecol..

[15] Burkle L.A., Mihaljevic J.R., Smith K.G. (2012). Effects of an invasive plant transcend ecosystem boundaries through a dragonfly-mediated trophic pathway. Oecologia.

[16] Chou A., Lin C., Cronin T.W. (2020). Visual metamorphoses in insects and malacostracans: Transitions between an aquatic and terrestrial life. Arthropod Struct. Dev..

[17] Rowe R.J. (2003). Review of “Dragonflies: Behaviour and Ecology of Odonata” by P.S. Corbet. Aust. J. Entomol..

[18] Corbet P.S. (1999). Dragonflies: Behaviour and Ecology of Odonata.

[19] Bastos R.C., Brasil L.S., Oliveira-Junior J.M.B., Carvalho F.G., Lennox G.D., Barlow J., Juen L. (2021). Morphological and phylogenetic factors structure the distribution of damselfly and dragonfly species (Odonata) along an environmental gradient in Amazonian streams. Ecol. Indic..

[20] Béthoux O., Anderson J.M. (2023). New light shed on Triadophlebiomorpha wing morphology and systematics (Insecta: Odonata). Geodiversitas.

[21] Corbet P.S., Suhling F., Soendgerath D. (2006). Voltinism of Odonata: A review. Int. J. Odonatol..

[22] Kalkman V.J., Clausnitzer V., Dijkstra K.-D.B., Orr A.G., Paulson D.R., van Tol J. (2008). Global diversity of dragonflies (Odonata) in freshwater. Hydrobiologia.

[23] Dumont H.J., Vierstraete A., Vanfleteren J.R. (2010). A molecular phylogeny of the Odonata (Insecta). Syst. Entomol..

[24] Walia G.K., Dhillon G.K. (2023). Phylogenetic analysis of *Aciagrion* Selys (Odonata: Coenagrionidae). J. Nat. Hist..

[25] Dijkstra K.-D.B., Bechly G., Bybee S.M., Dow R.A., Dumont H.J., Fleck G., Garrison R.W., Hämäläinen M., Kalkman V.J., Karube H. (2013). The classification and diversity of dragonflies and damselflies (Odonata). In: Zhang, Z.-Q. (Ed.) Animal Biodiversity: An Outline of Higher-Level Classification and Survey of Taxonomic Richness (Addenda 2013). Zootaxa.

[26] Geraldo de Carvalho F., Duarte L., Nakamura G., Dubal dos Santos Seger G., Juen L. (2021). Changes of phylogenetic and taxonomic diversity of Odonata (Insecta) in response to land use in Amazonia. Forests.

[27] Wellenreuther M., Svensson E.I., Hansson B. (2014). Sexual selection and genetic colour polymorphisms in animals. Mol. Ecol..

[28] Futahashi R. (2016). Color vision and color formation in dragonflies. Curr. Opin. Insect Sci..

[29] Okude G., Futahashi R. (2021). Pigmentation and color pattern diversity in Odonata. Curr. Opin. Genet. Dev..

[30] Bried J.T., Samways M.J. (2015). A review of odonatology in freshwater applied ecology and conservation science. Freshw. Sci..

[31] Oliveira-Junior J.M.B., Rocha T.S., Vinagre S.F., Miranda-Filho J.C., Mendoza-Penagos C.C., Dias-Silva K., Juen L., Calvão L.B. (2022). A bibliometric analysis of the global research in Odonata: Trends and gaps. Diversity.

[32] Miguel T.B., Oliveira-Junior J.M.B., Ligeiro R., Juen L. (2017). Odonata (Insecta) as a tool for the biomonitoring of environmental quality. Ecol. Indic..

[33] Palacino-Rodríguez F. (2016). Two decades of progress in over one hundred years of study: Present status of Odonata research in Colombia. Odonatologica.

[34] Chen C. (2006). CiteSpace II: Detecting and visualizing emerging trends and transient patterns in scientific literature. J. Am. Soc. Inf. Sci. Technol..

[35] Chen C., Ibekwe-SanJuan F., Hou J. (2010). The structure and dynamics of cocitation clusters: A multiple-perspective cocitation analysis. J. Am. Soc. Inf. Sci. Technol..

[36] Brandes U. (2001). A faster algorithm for betweenness centrality. J. Math. Sociol..

[37] Kleinberg J. (2003). Bursty and hierarchical structure in streams. Data Min. Knowl. Discov..

[38] Egghe L. (2006). Theory and practise of the g-index. Scientometrics.

[39] Monteiro Júnior C.d.S., Juen L., Hamada N. (2015). Analysis of urban impacts on aquatic habitats in the central Amazon basin: Adult odonates as bioindicators of environmental quality. Ecol. Indic..

[40] Clausnitzer V., Kalkman V.J., Ram M., Collen B., Baillie J.E.M., Bedjanič M., Darwall W.R.T., Dijkstra K.-D.B., Dow R., Hawking J. (2009). Odonata enter the biodiversity crisis debate: The first global assessment of an insect group. Biol. Conserv..

[41] Dijkstra K.-D.B., Kalkman V.J., Dow R.A., Stokvis F.R., van Tol J. (2014). Redefining the damselfly families: A comprehensive molecular phylogeny of Zygoptera (Odonata). Syst. Entomol..

[42] Stoks R., Córdoba-Aguilar A. (2012). Evolutionary ecology of Odonata: A complex life cycle perspective. Annu. Rev. Entomol..

[43] Svensson E.I., Abbott J., Härdling R. (2005). Female polymorphism, frequency dependence, and rapid evolutionary dynamics in natural populations. Am. Nat..

[44] Bybee S.M., Ogden T.H., Branham M.A., Whiting M.F. (2008). Molecules, morphology and fossils: A comprehensive approach to odonate phylogeny and the evolution of the odonate wing. Cladistics.

[45] Oliveira-Junior J.M.B.D., Shimano Y., Gardner T.A., Hughes R.M., de Marco Júnior P., Juen L. (2015). Neotropical dragonflies (Insecta: Odonata) as indicators of ecological condition of small streams in the eastern Amazon. Austral Ecol..

[46] Oliveira-Junior J.M.B., Juen L. (2019). The Zygoptera/Anisoptera Ratio (Insecta: Odonata): A New Tool for Habitat Alterations Assessment in Amazonian Streams. Neotrop. Entomol..

[47] Kalkman V.J., Boudot J.-P., Bernard R., De Knijf G., Suhling F., Termaat T. (2018). Diversity and conservation of European dragonflies and damselflies (Odonata). Hydrobiologia.

[48] Oliveira-Junior J.M.B.D., De Marco Junior P., Dias-Silva K., Leitão R.P., Leal C.G., Pompeu P.S., Gardner T.A., Hughes R.M., Juen L. (2017). Effects of human disturbance and riparian conditions on Odonata (Insecta) assemblages in eastern Amazon basin streams. Limnologica.

[49] Bybee S., Córdoba-Aguilar A., Duryea M.C., Futahashi R., Hansson B., Lorenzo-Carballa M.O., Schilder R., Stoks R., Suvorov A., Svensson E.I. (2016). Odonata (dragonflies and damselflies) as a bridge between ecology and evolutionary genomics. Front. Zool..

[50] Bowler D.E., Eichenberg D., Conze K.-J., Suhling F., Baumann K., Benken T., Bönsel A., Bittner T., Drews A., Günther A. (2021). Winners and losers over 35 years of dragonfly and damselfly distributional change in Germany. Divers. Distrib..

[51] Raebel E.M., Merckx T., Riordan P., Macdonald D.W., Thompson D.J. (2010). The dragonfly delusion: Why it is essential to sample exuviae to avoid biased surveys. J. Insect Conserv..

[52] Garrison R.W., Ellenrieder N.V., Louton J.A. (2010). Damselfly Genera of the New World: An Illustrated and Annotated Key to the Zygoptera.

[53] Dijkstra K.-D.B., Lewington R. (2006). Field Guide to the Dragonflies of Britain and Europe.

[54] McPeek M.A. (1998). The consequences of changing the top predator in a food web: A comparative experimental approach. Ecol. Monogr..

[55] Thomas C.D., Cameron A., Green R.E., Bakkenes M., Beaumont L.J., Collingham Y.C., Erasmus B.F.N., de Siqueira M.F., Grainger A., Hannah L. (2003). Extinction risk from climate change. Nature.

[56] Bomphrey R.J., Nakata T., Henningsson P., Lin H.-T. (2016). Flight of the dragonflies and damselflies. Phil. Trans. R. Soc. B.

[57] Appel E., Heepe L., Lin C.-P., Gorb S.N. (2015). Ultrastructure of dragonfly wing veins: Composite structure of fibrous material supplemented by resilin. J. Anat..

[58] Eagles-Smith C.A., Willacker J.J., Nelson S.J., Pritz C.M.F., Krabbenhoft D.P., Chen C.Y., Ackerman J.T., Grant E.H.C., Pilliod D.S. (2020). A national-scale assessment of mercury bioaccumulation in United States national parks using dragonfly larvae as biosentinels through a citizen-science framework. Environ. Sci. Technol..

[59] Maneechan W., Prommi T.O. (2022). Occurrence of microplastics in edible aquatic insect *Pantala* sp. (Odonata: Libellulidae) from rice fields. PeerJ.

